# Ketamine Treatment for Alcohol Use Disorder: A Review

**DOI:** 10.1192/j.eurpsy.2025.976

**Published:** 2025-08-26

**Authors:** B. Peixoto, M. Cruz, V. Ustares

**Affiliations:** 1 Psychiatry, Hospital do Divino Espírito Santo, Ponta Delgada, Portugal

## Abstract

**Introduction:**

Alcohol Use Disorder (AUD) is a pervasive condition with substantial psychological, social, and physical consequences. Traditional treatment approaches often struggle with high relapse rates, highlighting the need for more effective interventions. Recently, ketamine, an N-methyl-D-aspartate (NMDA) receptor antagonist, has emerged as a potential treatment for AUD due to its unique pharmacological properties.

**Objectives:**

The aim of this study is to evaluate the current evidence of ketamine treatment for alcohol use disorder and its efficacy.

**Methods:**

The authors did a non-systematic review of the current literature.

**Results:**

The results suggest that ketamine combined with psychotherapy reduces alcohol consumption and prolongs abstinence in AUD patients. The mechanism is hypothesized to involve ketamine’s ability to enhance neuroplasticity and modulate glutamatergic pathways, which may improve motivation and cognitive control. Additionally, ketamine’s rapid antidepressant effects could address comorbid conditions like depression and anxiety, often observed in AUD patients, thereby reducing the overall risk of relapse.

**Conclusions:**

Ketamine presents a promising adjunct to existing therapies for AUD, offering benefits that extend beyond traditional treatment approaches. However, while preliminary findings are encouraging, further research is necessary to confirm the long-term safety and efficacy of ketamine in treating AUD, particularly concerning optimal dosing strategies and the integration with psychotherapeutic interventions.

**Disclosure of Interest:**

None Declared

